# Supplementary material for: Curated and harmonised transcriptomics datasets of interstitial lung diseases
Source: Data Brief. 2025 Oct 14;63:112139. doi: 10.1016/j.dib.2025.112139 (PMC12581653; doi:10.1016/j.dib.2025.112139)
Supplement: Supplementary file 1 [file mmc1.zip › Supplementary_material/DNA-microarray/GSE10667/GSE10667_eUTOPIA_Agilent_QC_Report_2024-02-15.tar.gz!/Users/OWNER/AppData/Local/Temp/RtmpspbZX7/2024-02-15/msd.pdf]

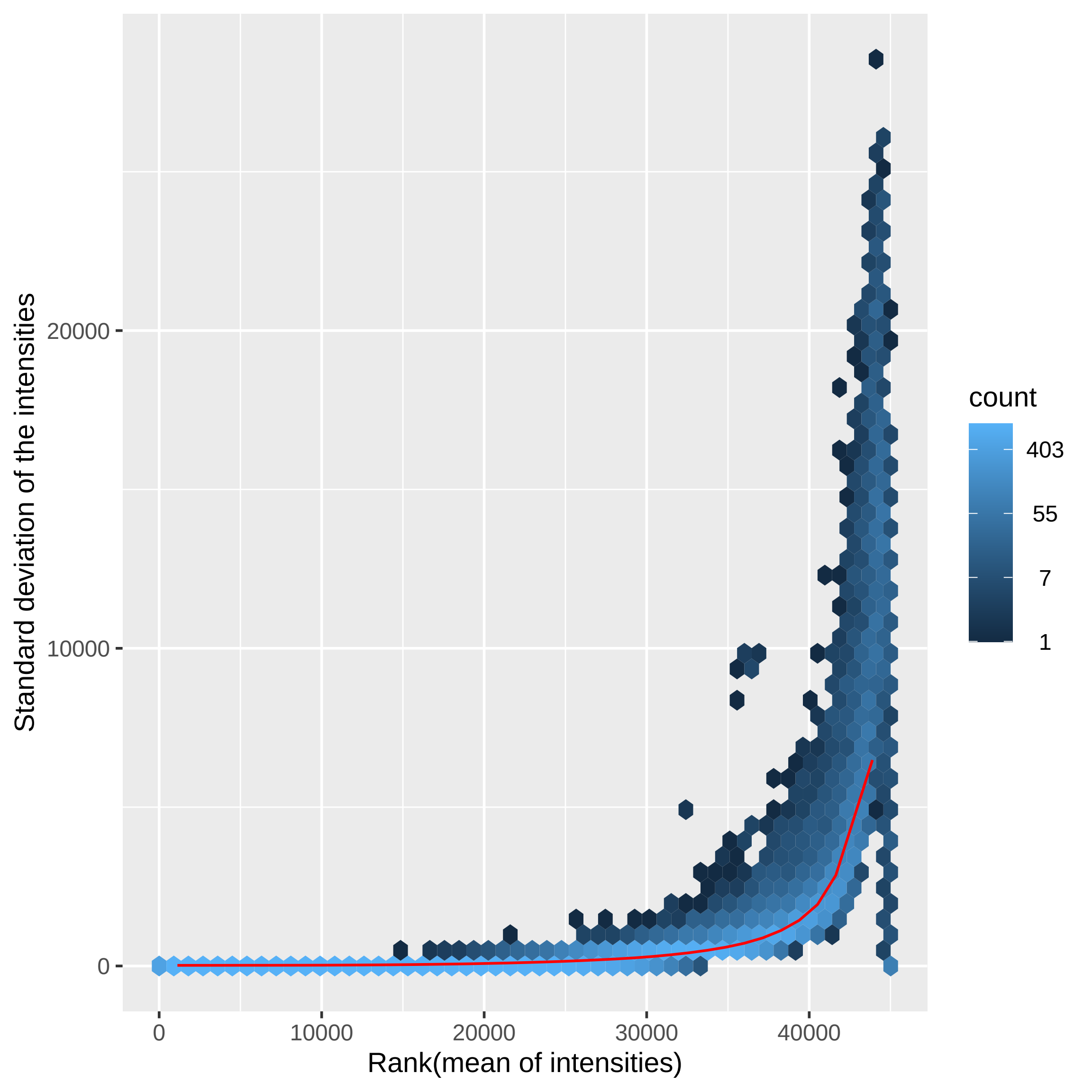

Standard deviation of the intensities
20000
10000
0
0
10000
20000
30000
40000
Rank(mean of intensities)
count
403
55
7
1
